# Prediction of the immunological and prognostic value of five signatures related to fatty acid metabolism in patients with cervical cancer

**DOI:** 10.3389/fonc.2022.1003222

**Published:** 2022-11-03

**Authors:** Qiongjing Zeng, Huici Jiang, Fang Lu, Mingxu Fu, Yingying Bi, Zengding Zhou, Jiajing Cheng, Jinlong Qin

**Affiliations:** ^1^ Department of Obstetrics and Gynecology, Shanghai Fourth People’s Hospital, School of Medicine, Tongji University, Shanghai, China; ^2^ Department of Burn Surgery, Ruijin Hospital, School of Medicine, Shanghai Jiao Tong University, Shanghai, China

**Keywords:** prediction, immunological and prognostic value, five signatures, acid metabolism, cervical cancer

## Abstract

A growing attention has been attached to the role of fatty acid metabolism (FAM) in the development of cancer, and cervical cancer (CC) is still the primary cause of cancer-associated death in women worldwide. Therefore, it is imperative to explore the possible prognostic significance of FAM in CC. In this study, CC samples and corresponding normal samples were acquired from the Cancer Genome Atlas (TCGA) and Genotype-Tissue Expression (GTEx). Single sample gene set enrichment analysis (ssGSEA) was conducted for calculating FAM-related scores (FAMRs) to screen FAM-related genes (FAMRGs). Two subtypes related to FAM were identified by consistent clustering. Among them, subtype C2 had a poor prognosis, and C1 had a high level of immune cell infiltration, while C2 had a high possibility of immune escape and was insensitive to chemotherapy drugs. Based on the differentially expressed genes (DEGs) in the two subtypes, a 5-gene signature (PLCB4, FBLN5, TSPAN8, CST6, and SERPINB7) was generated by the least absolute shrinkage and selection operator (LASSO) and Akaike information criterion (AIC). The model demonstrated a high prognostic accuracy (area under the curve (AUC)>0.7) in multiple cohorts and was one independent prognostic factor for CC patients. Accordingly, FAMRGs can be adopted as a biomarker for the prediction of CC patients’ prognosis and help guide the immunotherapy of CC.

## Introduction

Since the World Health Organization (WHO) called for the worldwide elimination of cervical cancer (CC) in 2018, various preventive measures for CC have emerged one after another, among which human papillomavirus (HPV) vaccine and cervical screening are the two most effective interventions (1). However, such prevention and treatment schemes are extremely limited by resources and basic health facilities, and the coverage of them in low-and middle-income countries is less than one tenth of that in developed countries, so CC is still the primary cause of cancer-associated death in poor countries over the globe (2, 3). At present, there is still a need to develop a brand-new screening technology that can identify the symptoms in the incubation period and early stage of CC, and is affordable in most regions, thus reducing the difference in the incidence of CC worldwide due to the gap in resources and infrastructure by greatly lowering the incidence of CC in developing countries (4, 5). Therefore, a faster and more cost-effective screening method for CC is still wanted worldwide (6).

As the next-generation sequencing technology and the accumulation of CC sequencing data develop, it becomes clear and cost-effective to find biomarkers for prognosis assessment and treatment of CC through genome-wide analysis (7). Valuable decision-making guidance can be provided for clinicians by unbiased synthesis of various data, screening of molecular characteristics of cancer-causing subgroups of CC, and re-classification of them, so that more medical resources can be concentrated on high-risk CC patients who really have disease progression, and the economic and psychological burden caused by HPV vaccination and cervical screening can be greatly reduced (7–9).

Compared with normal cells, tumor cells often have different cell metabolic phenotypes to meet the energy needs of rapid cell proliferation and growth (10, 11). Recently, a growing number of studies have found that lipid metabolism disorder often occurs in the development of various human malignant tumors including prostate cancer (12) and colon cancer (13), and the change of FAM has greatly promoted the energy conversion of cancer cells (14). All the activities of tumor cells are inseparable from the intake and synthesis of fatty acids (15). The gradually accumulated fatty acids seem to be bound up with the disease recurrence and unfavorable prognosis of patients, and the metabolic characteristics of fatty acids may become a new target of anti-cancer therapy (12, 14, 15).

Zhang et al. (16) have found that fatty acid-binding protein 5 induces lymphatic metastasis of CC through metabolic reprogramming, but the clinical significance of FAM-related characteristics in CC is still under investigation, and it is still a challenge to identify stable fatty acid-related signature. This study comprehensively analyzed the expression, immune and prognostic characteristics of fatty acid metabolism-related genes (FAMRGs) in CC, identified two different CC subtypes associated with FAM and their immune characteristics, and verified the FAM-associated prognosis model by multiple cohorts, which provided a theoretical basis for forecasting the survival risk of CC patients.

## Methods

### Variation analysis acquisition and pre-processing of data sets

From the Cancer Genome Atlas (TCGA, https://www.cancer.gov/about-nci/organization/ccg/research/structural-genomics/tcga) (17) and Genotype-Tissue Expression (GTEx) (https://commonfund.nih.gov/gtex) (18), the data about expression profile of CC tissues and normal cervical tissues were downloaded. Their batch effect was eliminated by the remove batch effect function of limma in the R package, and two data sets were corrected by the normalize between arrays function. Principal Component Analysis (PCA) was used for evaluating the degree of batch effect removal. Totally 300 data of CC expression profiles were downloaded from the GSE44001 dataset of Gene Expression Omnibus (GEO) as a verification set (19), and files of the probe platform were downloaded. The probe ID numbers were annotated to gene symbols. Probes corresponding to multiple genes meantime were removed, and the value of probes with the same gene expression was averaged.

In addition, the 272 tumour samples in TCGA were assigned to a training set and a verification set in the random manner based on the proportion of 1:1 after 100 times of random grouping with replacement to facilitate the subsequent model construction.

Limma in the R package was adopted in the variation analysis of different groups, and the differentially expressed genes (DEGs) were screened with the absolute value of log_2_ (fold change) > log_2_ (1.2) and FDR< 0.05.

### Single-sample gene-set enrichment analysis

The fatty acid metabolism-related scores (FAMRs) were calculated through ssGSEA and R package GSVA after downloading the FAMRGs sets in the molecular signature database (MSigDb, c2.cp.kegg. v7.4.symbols) (20). The rcorr function in Hmisc in the R package was adopted for determining the correlation of FAMRs with DEGs. The correlation with FAMRGs was found with cor > 0.2 and FDR<0.05 as the filter condition.

### Survival analysis

Univariate Cox analysis was carried out by the coxph function of Survival in the R package to screen the genes associated with CC patients’ prognosis, with p<0.05 as the filter condition. The log rank test was adopted for analyzing the survival differences between groups and corresponding Kaplan-Meier (K-M) curves were drawn.

### Construction of FAM-related subtypes

272 CC samples were consistently clustered using ConsensusClusterPlus in the R package, and 500 times of bootstraps were performed by the pam algorithm and “Pearson” as the measurement distance. Each bootstrap process covered 80% of patients in the training set. With the number of clusters set to 2 to 10, the consistency matrix and consistency cumulative distribution function were calculated for determining the optimal classification.

### Analysis of immune escape characteristics

According to the previous research (21, 22), the molecular characterization of aneuploid score, nosilent mutation rate, fraction altered, number of segments, and homologous recombination defects were collected to evaluate tumour immunogenicity among different subtypes, and maftools in the R package was used for visually analyzing the mutation data of the top 10 genes with significant differences in expression.

### Calculation of the difference in immune microenvironment among different subtypes

The CIBERSORT algorithm in IOBR of the R package was adopted for calculating the relative abundance of 22 kinds of immune cells in CC (23), and the ESTIMATE algorithm was adopted for calculating the matrix score and immune score of each sample of CC (24).

### Prediction of clinical efficacy

With the Tumour Immune Dysfunction and Exclusion (TIDE) algorithm developed by Jiang et al. (25), TIDE, IFNG, Dysfunction, Exclusion, and TAM.M2 scores were downloaded from TIDE (http://tide.dfci.harvard.edu) for predicting the clinical treatment response of different subtypes, and the Wilcox.test was used for comparing the scores among different subtypes. Additionally, the half-maximum inhibitory concentration (IC50) of traditional drugs was downloaded from Genomics of Drug Sensitivity in Cancer (GDSC, https://www.cancerrxgene.org/) (26), and pRRophetic in the R packet was used for predicting the chemotherapy response of CC samples.

### Construction of prognosis-related signature based on FAMRGs

The glmnet in the R package was used for further feature selection by the least absolute shrinkage and selection operator (LASSO), and a risk model was built by 10-fold cross-validation. According to Akaike information criterion (AIC), the complexity of the model was evaluated, and the number of parameters was gradually deleted to acquire the optimal model. The RiskScore of patients with different subtypes was calculated ( $\text{RiskScore}=\sum_{i=1}^{n}Coef(i)*Exp(i)\;$ ), and Coef was taken as the characteristic coefficient of each signature. Exp presented the expression of each signature in CC samples. The samples of RiskScore with Z score and RiskScore > 0 were assigned to a high-risk group and those with scores<0 to a low-risk group, and the timerROC in the R package was used to evaluate the prediction accuracy of different risk levels. The rms in the R package was adopted for establishing nomograms to predict the1-year, 3-year and 5-year overall survival rates and calculate the prognosis risk of individual patients. The Decision Curve Analysis (DCA) curve was drawn by ggDCA in the R-packet for evaluating the clinical predictive performance of the model.

### Clinical sample collection and qPCR validation

100 cases of cervical cancer tissues and 100 cases of adjacent tissues in our hospital were collected, and qPCR verification of PLCB4, FBLN5, TSPAN8, CST6, and SERPINB7 genes was performed. The tissue samples were fully ground with liquid nitrogen, 1 ml of Trizol (Invitrogen) solution was added, mixed well, and placed at room temperature for 5 minutes to fully lyse; (the sample name should be marked on the tube cover and tube wall) qPCR verification was carried out according to the specific operation steps of qPCR. Primers: PLCB4, F:ACAGATACGAGGAGGAATCC, R: TCCATGTCAGAAAGAAGCC; FBLN5, F: CATCAATACTGAAGGCGGG, R: TCATCAATGTCTAAGCACTGG; TSPAN8, F: CAAGAAGAGTTTAAATGCTGCG, R: AGGCACATAATTCAGGATAGTG; CST6, F: TACTACTTCCGAGACACGC, R: AGGAAGTACTTGATGCCGG; SERPINB7, F: TCCCACAAGGATTATGATCTCAG, R: CTCAATGTAGTCCTTATGAAAGCC. The relative expression levels of PLCB4, FBLN5, TSPAN8, CST6 and SERPINB7 genes were calculated by 2^-△△CT^.

## Results

### Screening of FAMRGs

**Figure 1:**
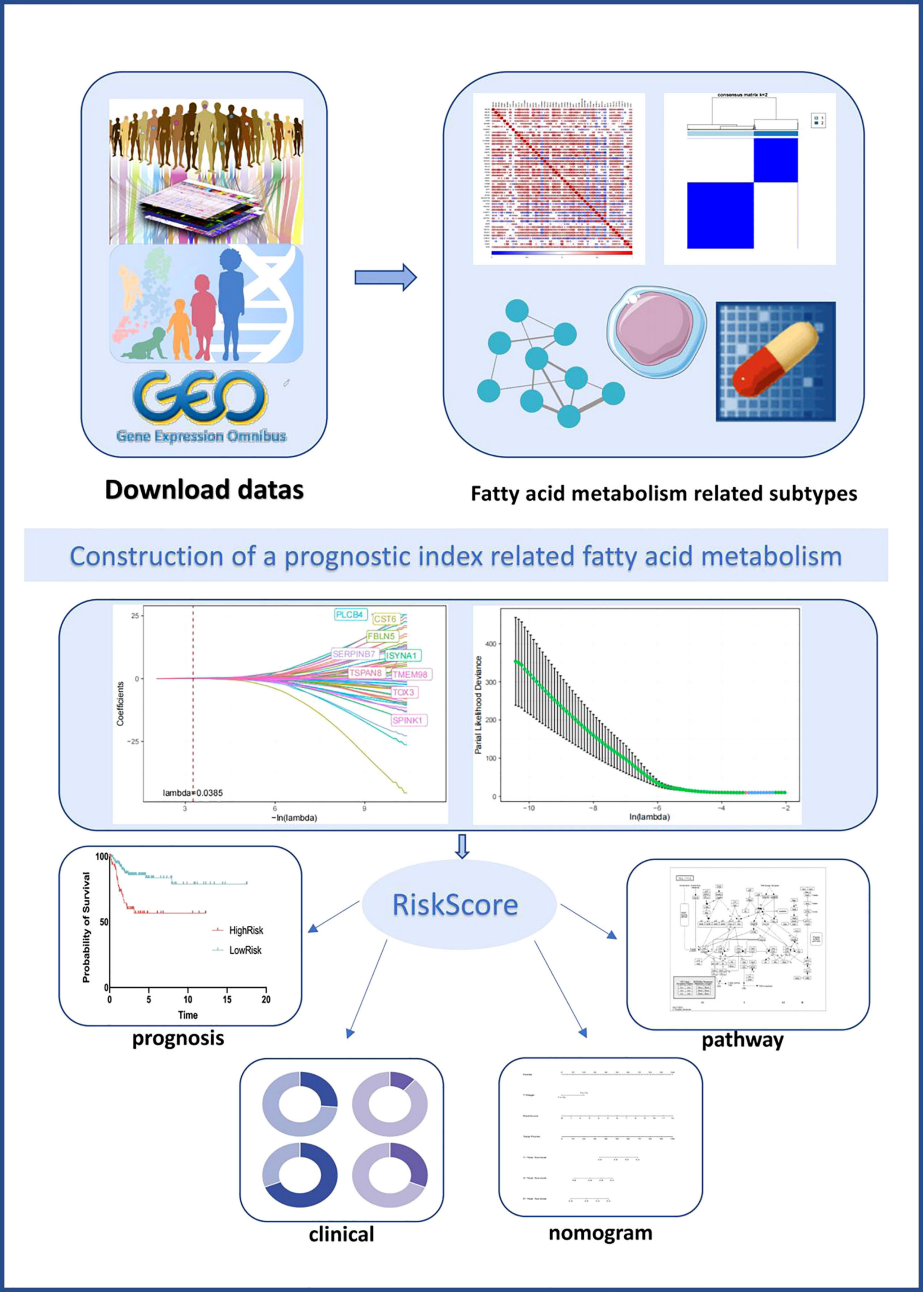
Graphical abstract of the construction of a prognostic index associated with fatty acid metabolism in cervical cancer.

**Figure 2:**
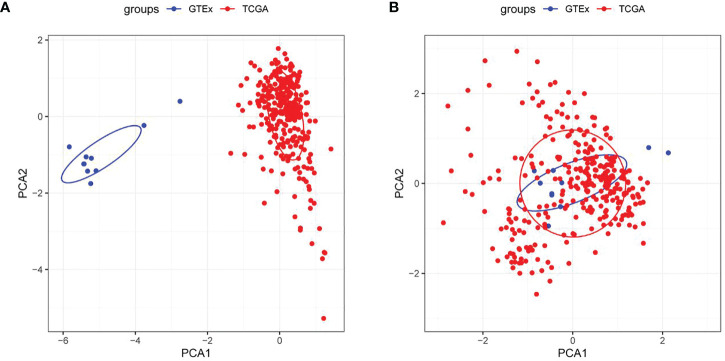
Evaluation of sample clustering by the principal component analysis (PCA) **(A)** PCA diagram between two data sets before the batch effect was removed; **(B)** PCA diagram between two data sets after the batch effect was removed. TCGA, The Cancer Genome Atlas; GTEx, Genotype-Tissue Expression.

The working route of this study is shown in **Figure 1**. According to PCA, two data sets were clustered together mainly according to their sources (**Figure 2A**), but after the integration of these data sets, the samples in the two data sets were mixed, and the batch effect between the data sets was eliminated (**Figure 2B**).

**Figure 3:**
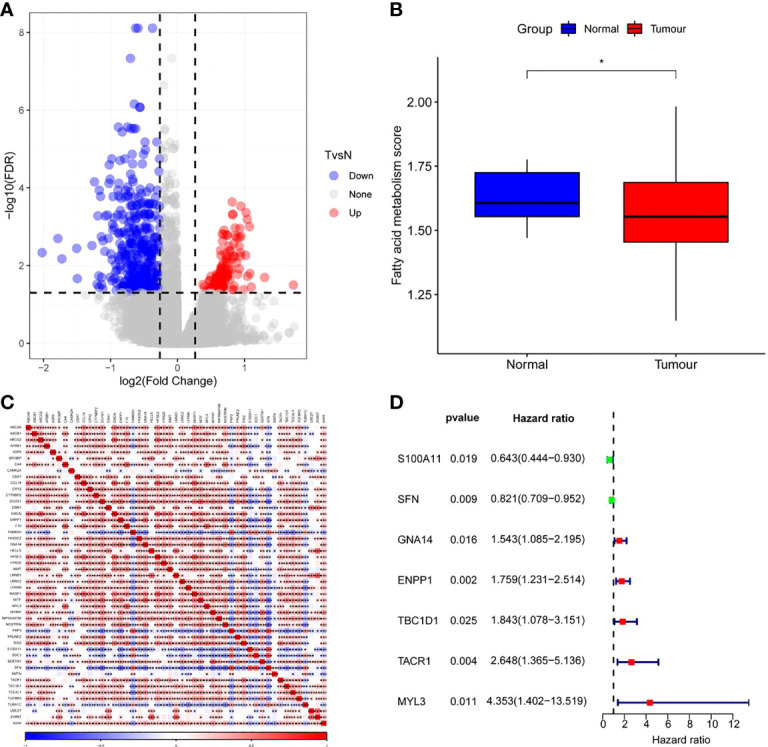
Screening of fatty acid metabolism-related genes (FAMRGs) **(A)**Volcano map of variation analysis between CC samples and normal samples; **(B)** Comparison of fatty acid metabolism-related scores (FAMRs) between CC samples and corresponding normal samples; **(C)** FAMRGs-related Heat map, **(D)** Forest map of prognosis-related FAMRGs. * vs p<0.05. *p<0.05,**p<0.01,***p<0.001.

As shown in **Figure 3A**, 487 DEGs were selected from tumor samples of CC and corresponding normal samples, of which 120 DEGs were up-regulated and 367 DEGs were down-regulated. Furthermore, ssGSEA results revealed notably fewer FAMRGs in tumor samples than those in normal samples (**Figure 3B**) and also revealed differences in FAM between CC tissues and normal tissues. Among them, 48 DEGs were greatly associated with FAM (**Figure 3C**). Univariate Cox analysis showed that 7 FAMRGs including S100A11 were bound up with the prognosis of CC patients (**Figure 3D**; **Supplementary Table 1**).

### Identification of two different FAM-related subtypes based on FAMRG

**Figure 4:**
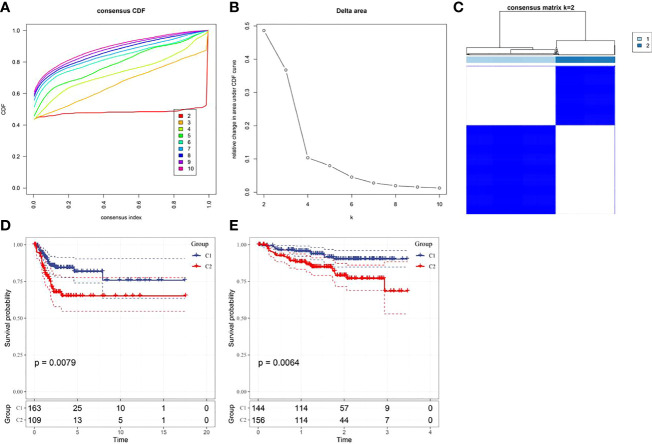
Construction of fatty acid metabolism-related subtypes and prognosis **(A)** Consensus clustering samples between each category number k in the TCGA cohort; **(B)** Delta area curve of cumulative distribution function (CDF) of TCGA cohort sample; **(C)** Heat map of sample clustering when k=2; **(D)** Kaplan-Meier curve of two subtypes in the TCGA cohort; **(E)** The prognostic Kaplan-Meier curves of the two subtypes in the GSE44001 cohort.

**Figure 5:**
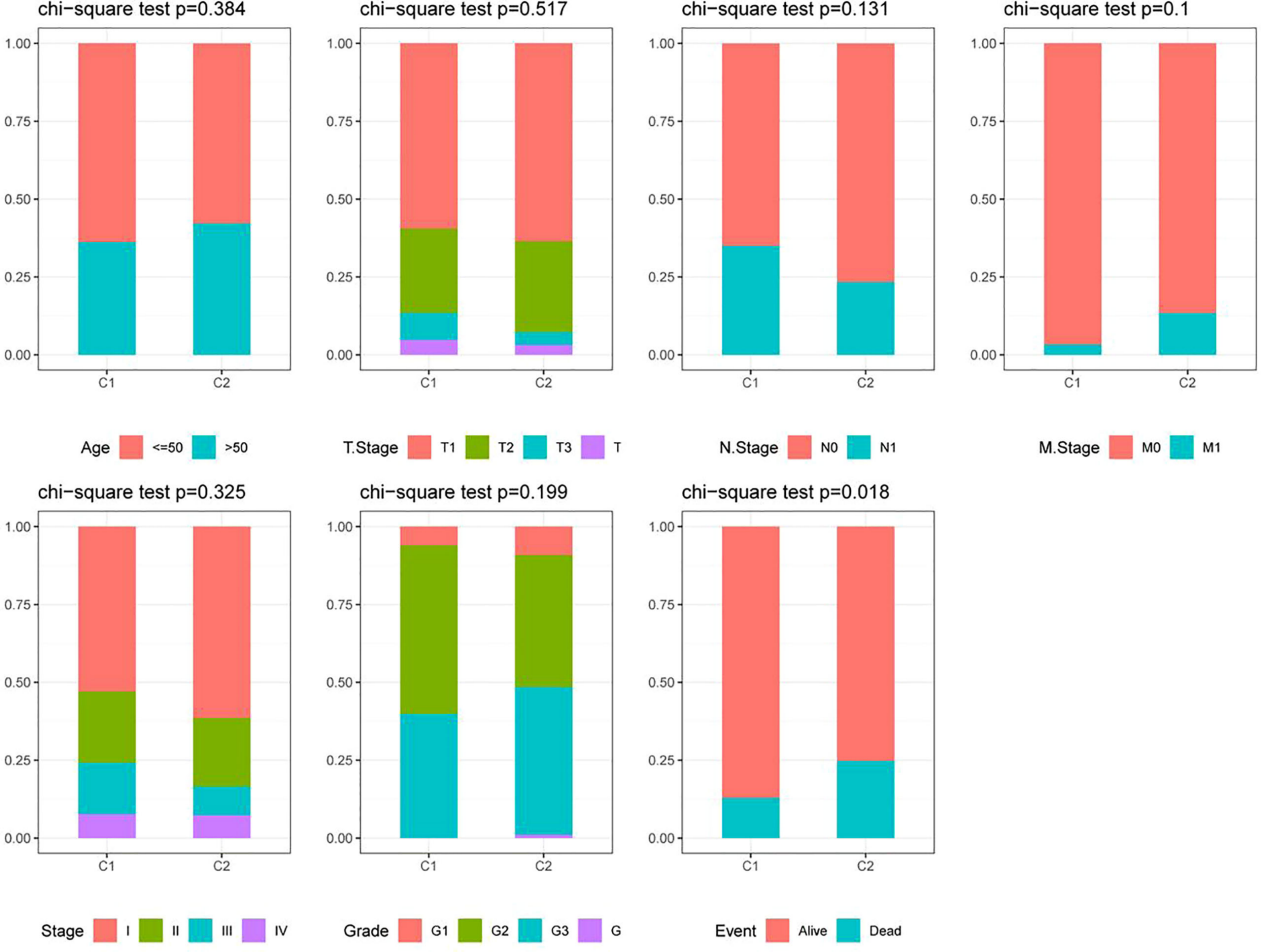
Distribution of clinicopathological features between two fatty acid metabolism-related subtypes in the TCGA cohort.

Based on the cumulative distribution function (CDF) and CDF Delta area curve, the optimal number of clusters (**Figures 4A, B**) was determined. When k=2, there was a comparatively stable clustering result, and two subtypes (C1, and C2) were obtained (**Figure 4C**). Further analysis of the prognosis of these two CC subtypes revealed a notably lower survival rate in patients from the C2 group that that in patients from the C1 group at the same time (p<0.05, **Figure 4D**). Similarly, the same difference in GSE44001 was found. The same method was adopted for processing the CC samples from GSE44001. Patients in Group C2 still had an unfavorable prognosis (**Figure 4E**), which was similar to the results of the data set from TCGA. The findings indicate that the two subtypes based on FAMRG can be transplanted in different research cohorts. The Chi-square test was used for comparing the distribution of different clinicopathological features between the two subtypes, and the results revealed notable differences in the living conditions of CC patients in the TCGA cohort between the two groups (**Figure 5**, p<0.05).

### Immune characteristics of FAM-related subtypes

**Figure 6:**
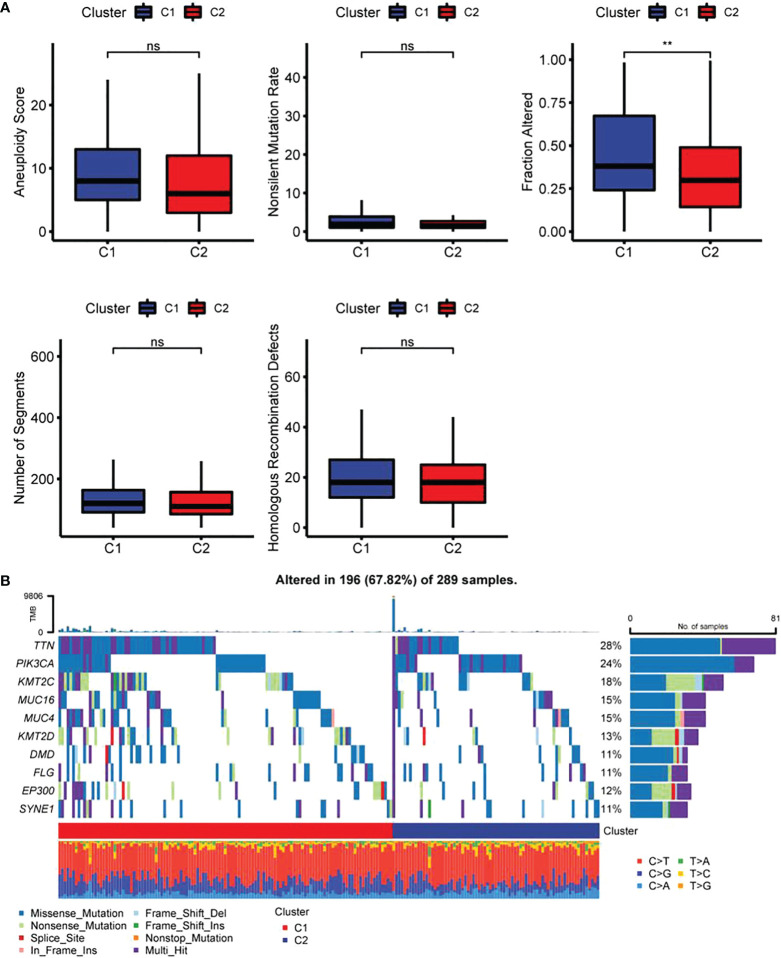
Differences in genome mutation between two fatty acid-related subtypes **(A)** Differences of Homologous Recombination Defects, Aneuploidy Score, Fraction Altered, Number of Segments and Tumor mutation burden in molecular subtypes in the TCGA cohort; **(B)** Somatic mutation landscape in two molecular subtypes. ** p<0.01, ns, P>0.05.

**Figure 7:**
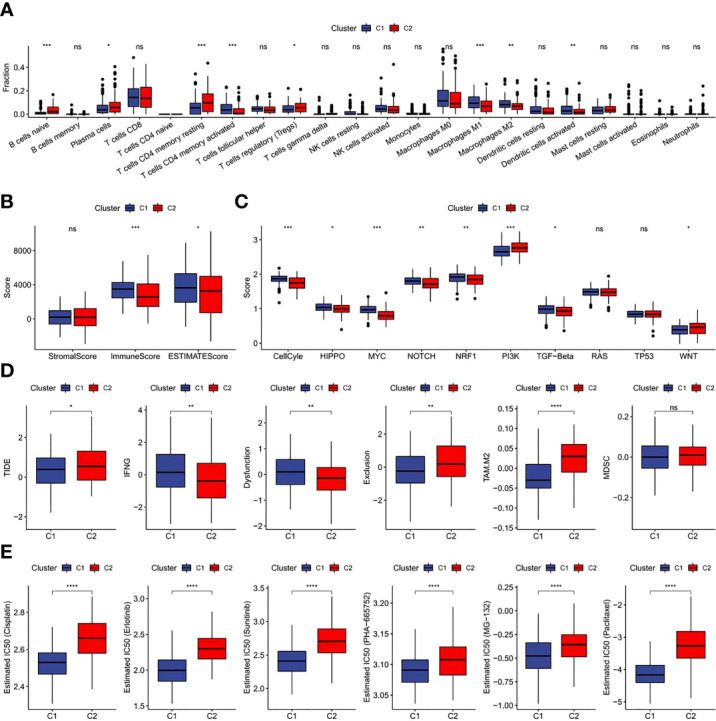
Immunological characteristics, immunotherapy, chemotherapy and target therapy responses of two fatty acid-related subtypes **(A)** Difference of 22 immune cell scores between different molecular subtypes in the TCGA cohort; **(B)** Difference of ESTIMATE immune infiltration between different molecular subtypes in the TCGA cohort; **(C)** Difference in the score of 10 pathways related to tumor abnormality between different subgroups in the TCGA cohort; **(D)** Difference of TIDE analysis results between different groups in the TCGA cohort; **(E)** The box plots of the estimated IC50 for drug in TCGA-CECS. *p<0.05,**p<0.01,***p<0.001, ****p<0.0001, ns, P>0.05.

The results revealed a notably higher fraction altered in C1 than that in C2 (**Figure 6A**), and also showed that genes with significant differences in CC such as TTN and PIK3CA had higher mutation frequency in C1 (**Figure 6B**). The potential function of FAM-related subtypes in CC was further analyzed, and the proportion of 22 immune cell types between the two subtypes was evaluated by CIBERSORT. Compared with C2, the proportion of B cells navie, Plasma cells, T cells memory resting and T cells regulatory (Tregs) in C1 was significantly lower, while T cells memory activated, Macrophages M1 and Dendritic cells activated were significantly enriched (**Figure 7A**). C1 got higher immune score and estimate score than C2, and C1 had a higher level of immune cell infiltration (**Figure 7B**). As shown in **Figure 7D**, the TIDE score of subtype C2 in the TCGA cohort was higher than that of C1, suggesting that subtype C2 was more likely to escape and less likely to benefit from immunotherapy. The IC50 of 6 traditional chemotherapeutic drugs in C1 was significantly lower than that in C2, and these drugs were more effective in C1 patients (**Figure 7E**).

### Construction and verification of prognosis-related model of FAMRGs

**Figure 8:**
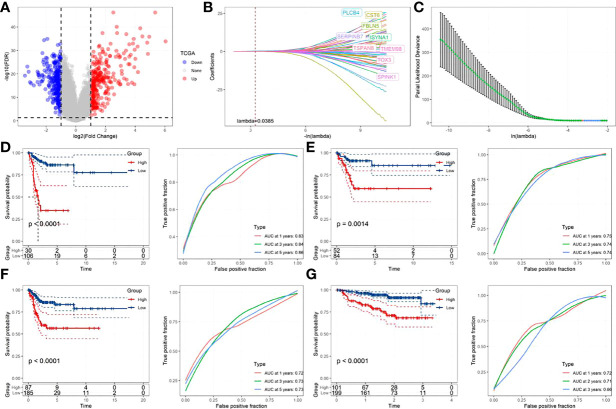
Construction and verification of prognostic correlation model of FamRGS **(A)**Volcanic map of the variation analysis of molecular subtypes; **(B)** The changing trajectory of each independent variable, with the horizontal axis representing the log value of the independent variable lambda, and the vertical axis representing the coefficient of the independent variable; **(C)** Confidence interval under each lambda; **(D)** AUC curve and KM curve of the risk model of the training set data from TCGA; **(E)** AUC curve and KM curve of the risk model of the verification set data from TCGA; **(F)** AUC curve and KM curve of the risk model of all data sets from TCGA; **(G)** AUC curve and KM curve of the risk model of data set from GSE44001.

Totally 558 DEGs of the two-fatty acid-related subtypes were screened by variation analysis (**Figure 8A**), and 58 DEGs related to prognosis were further filtered by univariate Cox analysis in the training set (**Supplementary Table 2**). When lambda= 0.0385, the model reached the optimal state (**Figures 8B, C**), and the parameters were further compressed to obtain a model composed of five genes: Riskscores=0.48×PLCB4+0.49×FBLN5+0.15×TSPAN8+0.38×CST6+0.30×SERPINB7 (See **Supplementary Table 3** for detailed descriptions of the genes.)

The Risk Score of each sample was calculated. As shown in **Figures 8D** and **E**, the training set and validation set of TCGA both revealed a shorter survival time in CC patients from the high-risk group than that from the low-risk one (p<0.05). Moreover, this model had high accuracy in the prediction and classification of CC in one year, three years and five years (area under the curve (AUC)>0.7). For further verifying the generalization ability of the model, all TCGA data and the independent data set GSE44001 were verified. The results, as shown in **Figures 8F** and **G**, were in agreement with those of the training set of TCGA. FAMRGs prognosis-associated model was a prognosis scoring system with high precision (AUC>0.7), and the high-risk group had an unfavorable prognosis.

### Association of RiskScore with other clinicopathological features and its prognostic value

**Figure 9:**
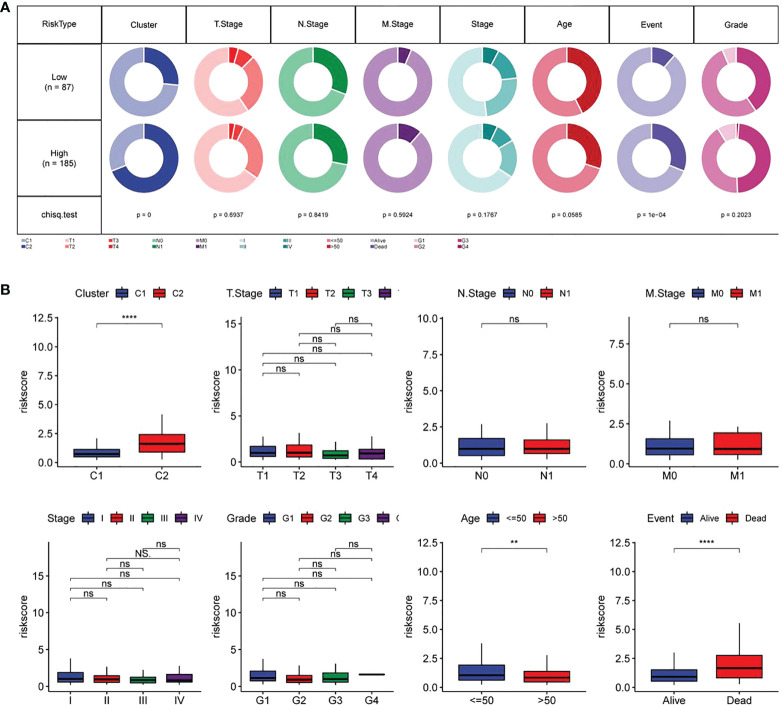
Relationship between RiskScore and different clinicopathological features of CC patients **(A)** Comparison in terms of the distribution of different clinical characteristics between high-and low-risk groups in TCGA data sets; **(B)** Differences in RiskScore of different clinical characteristics in TCGA data sets. **p<0.01, ****p<0.001, ns, P>0.05.

The associations of RiskScore with subtype, T. Stage, N. Stage, M. Stage, Stage, age, Event and Grade were tested. As shown in **Figure 9A**, the proportion of subtype C2 and dead population in the high-risk group was higher (p<0.05). Further comparison of the difference in RiskScore among people with different clinicopathological features revealed higher RiskScore in people with subtype C2, age ≤ 50 and death (**Figure 9B**).

**Figure 10:**
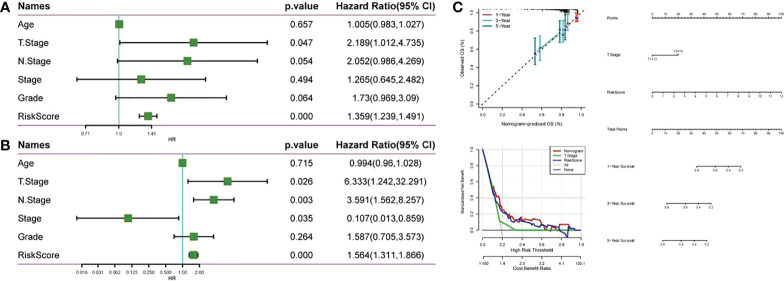
Compared with other clinicopathological features, RiskScore has higher prognostic value. **(A)** Forest map of clinical phenotype in the TCGA cohort and univariate Cox analysis of RiskScore; **(B)** Forest map of clinical phenotype in the TCGA cohort and multivariate Cox analysis of RiskScore; **(C)** Anomogram, nomogram alignment map and DCA curve constructed by TCGA data set.

Univariate and multivariate Cox regression analysis was used for evaluating the prognostic value of RiskScore and other clinicopathological characteristics in CC. As shown in **Figures 10A, B**, T. Stage and RiskScore were independent prognostic factors of CC patients, and RiskScore was the most significant prognostic factor. Then, a nomogram composed of T. Stage and RiskScore was constructed. According to **Figure 10C**, RiskScore made the greatest contribution to the survival prediction of CC patients. The nomogram correction map and DCA curve showed that RiskScore had higher predictive performance than other clinicopathological features.

### Biological pathway of potential regulation of FAMRGs prognosis-related model

**Figure 11:**
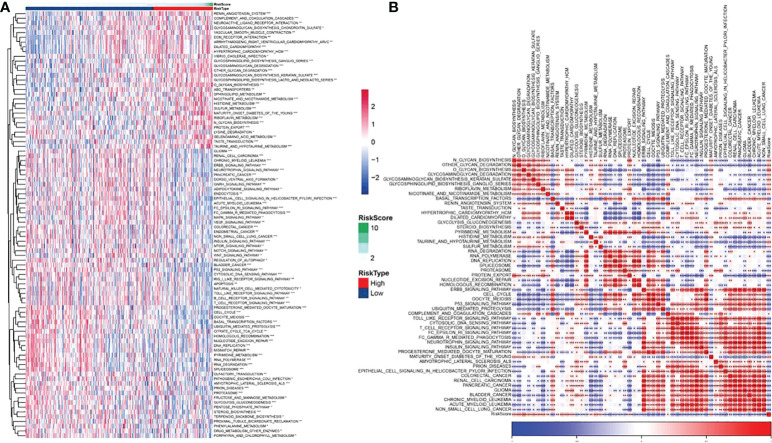
Biological pathway for potential regulation of FAMRGs prognosis-related model **(A)** Heat map in which GSVA analysis of TCGA dataset pathway showed significant enrichment scores of related pathways in high and low risk groups, and **(B)** Heat map of correlation analysis of pathways with significant differences in TCGA data sets and RiskScores (*p<0.05,**p<0.01,***p<0.001).

For better studying the potential function of the FAMRGs prognosis-related model, the score of each KEGG pathway in CC patients was calculated by GSVA package, and 90 significant pathways were calculated in the high-and low-risk groups (p< 0.05, **Supplement Table 3**), as shown in **Figure 11A**. Among them, there were 53 significant pathways in high and low risk groups (p< 0.001). The association of enrichment score with RiskScore was analyzed (**Figure 11B**; **Supplementary Table 4**). The FAMRGs prognosis-related model was significantly bound up with signals including O GLYCAN BIOSYNTHESIS, CELL CYCLE, BASAL TRANSCRIPTION FACTORS, and P53_SIGNALING_PATHWAY, which was similar to our previous research results (**Figure 7C**). There were significant differences in FAM-related subtypes among 10 classic oncogenic pathways (27), and FAMRGs prognosis-related model was strongly bound up with these signals.

### Clinical cohort qPCR validation

**Figure 12:**
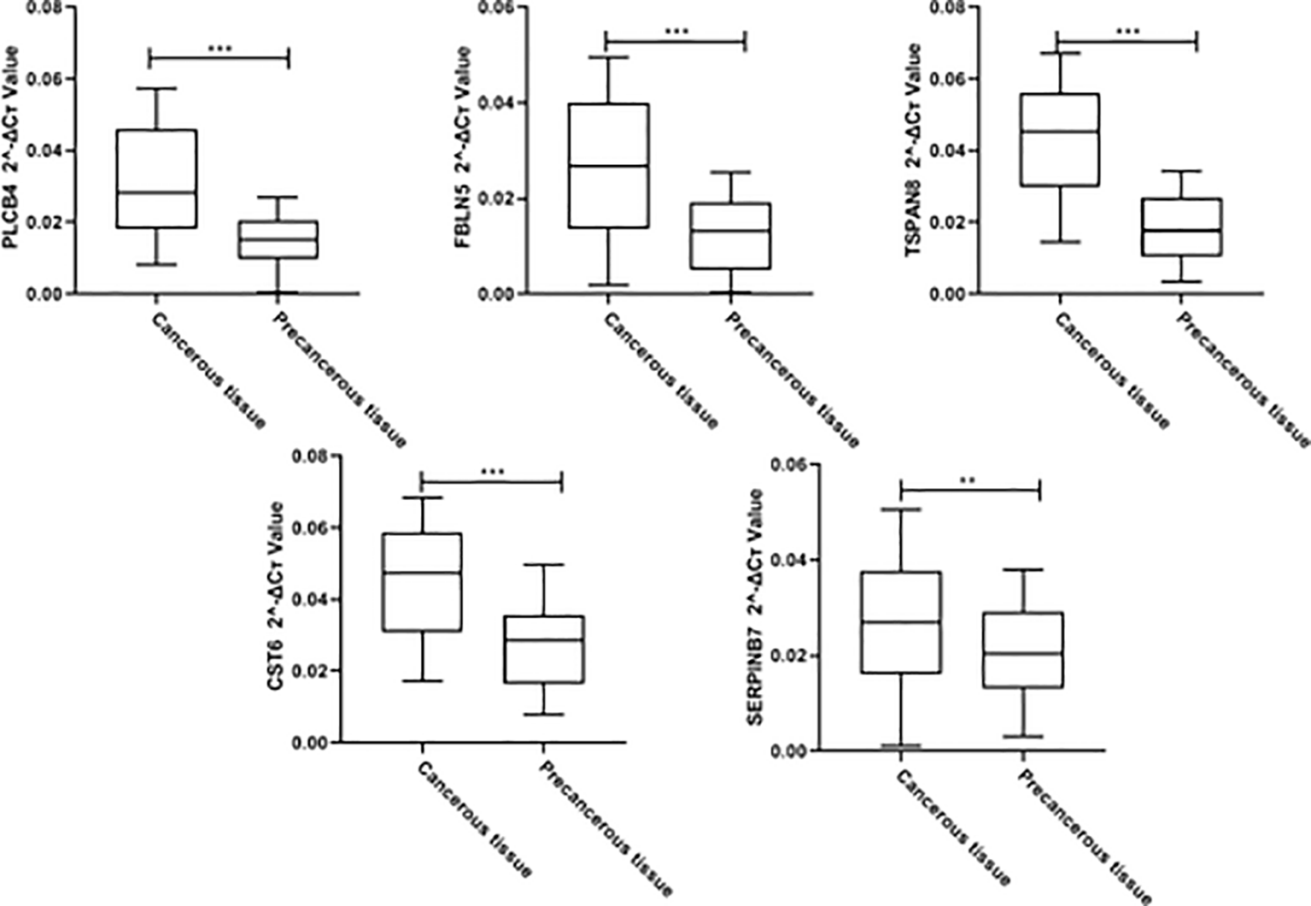
Clinical cohort qPCR validationClinical cohort qPCR validation for PLCB4, FBLN5, TSPAN8, CST6, and SERPINB7 (**p<0.01,*** p<0.001).

100 cervical cancer tissues and 100 paracancerous tissues were collected from our hospital for qPCR verification of PLCB4, FBLN5, TSPAN8, CST6, and SERPINB7. The results showed that PLCB4, FBLN5, TSPAN8, CST6, and SERPINB7 were highly expressed in cervical cancer tissues (**Figure 12**, p<0.05).

## Discussion

Compared with sugar metabolism and amino acid metabolism, FAM has received less attention, but the importance of fatty acids in the development of cancer is increasingly recognized (28, 29). As a crucial component of the membrane matrix, the fatty acid is a crucial messenger and fuel source for energy production (30). Compared with normal cells, tumour cells are more likely to rely on *de novo* synthesis to synthesize fatty acids for energy metabolism and membrane formation for the maintenance of the rapid growth and proliferation of cells (31). It is worth noting that there are many fatty acids and metabolic by-products of them, each of which has different feedback mechanisms and regulation nodes and affects many ways of disease behavior through extremely complex regulatory networks (32). Different tumor subtypes may drive specific lipid phenotypes (28, 31). Therefore, identifying a potential subtype related to FAM helps predict the CC patients’ prognosis.

By integrating cervical sample data from TCGA and GTEx, DEGs related to FAMRs were searched and molecular subtypes related to FAM (C1 and C2) were constructed. C2 showed poor prognosis in both TCGA cohort and GSE44001 cohort, independent verification set, and the proportion of deaths in subtype C2 was notably higher than that in subtype C1. In addition, a high Fraction of Altered was found in C1, and TTN and PIK3CA, common drivers in CC, have a high mutation frequency (14). C1 shows a higher proportion of T cells, macrophages and dendritic cells activated, and immune score and estimate score than C2, which means a lower level of tumor purity (24). The proportion of immune cells is high in the samples with lower tumor purity (33), and the inflammatory reaction caused by immune cells will increase the cell mutation rate and activate stronger anti-tumor characteristics and faster reaction speed (34, 35). The samples with higher tumor mutation load often show better immunotherapy effects (34, 36). These results were in agreement with our research results. The IC50 of traditional chemotherapeutic drugs in subtype C1 was notably lower than that in subtype C2, and subtype C1 was less likely to escape from immune surveillance than C2 and was more sensitive to immunotherapy.

Then, a prognostic model related to FAM was constructed for CC based on the two subtypes of DEGs, and the generalization and prediction accuracy of the model was repeatedly verified by multiple cohorts. Patients with subtype C2 and dead ones accounted for a higher proportion in the high-risk group, and these patients had a higher RiskScore. Consistent with our expectations, RiskScore can serve as one independent prognostic factor to predict the CC patients’ prognosis and contributes greatly to the prediction of the survival of CC. The accuracy of the model prediction has been further confirmed.

As we described above, the up regulation of FAM contributes to cell membrane production and signal transmission, including activation signals (4, 37). The enrichment of multiple signals is significantly different in different risk levels. Research has pointed out that one of the key mechanisms of signal transduction in CC cells is the glycosylation of proteins (38). As a glycoprotein on the cell surface, N-Glycon directly affects cell signal transduction and is the diagnostic target of malignant transformation in the early stage of CC (39–41). O-glycan can be used as a biological marker of proliferation, senescence and metastasis of CC cells by regulating immune response and controlling cell metabolism (42, 43). In addition, in the G2 phase, *de novo* synthesis is enhanced to synthesize lipids, which ensures the membrane material needed for mitosis and promotes cell proliferation (44). Subtype C1 had a higher score on cell cycle than subtype C2, and RiskScore and cell cycle enrichment showed a significantly negative correlation. The high-risk group and patients with subtype C2 may escape from the control of the cell cycle and fails, leading to continuous cell division and promoting cancer progress (45, 46).

Although some studies have explored biomarkers related to FAM in clear cell renal cell carcinoma (47) and bladder cancer (48), this study has revealed molecular subtypes related to FAM in CC for the first time and constructed a FAM-related prognostic model with strong predictive ability. It provides some new insights for accurate screening of CC, which is helpful to guide clinical treatment and prognosis prediction.

## Data availability statement

The original contributions presented in the study are included in the article/**Supplementary Material**. Further inquiries can be directed to the corresponding authors.

## Ethics statement

The studies involving human participants were reviewed and approved by the ethics committee of Shanghai Fourth People’s Hospital. The patients/participants provided their written informed consent to participate in this study.

## Author contributions

QZ, HJ, and FL are the mainly responsible for the writing of the article. YB and MF are mainly responsible for research design. ZZ, JC, and JQ are co-corresponding authors. They are responsible for ensuring that the descriptions are accurate and agreed by all authors. All authors contributed to the article and approved the submitted version.

## Funding

This study was supported by the Shanghai Science and Technology Commission/General Project of Natural Science Foundation of Shanghai in 2021(21ZR1440600).

## Conflict of interest

The authors declare that the research was conducted in the absence of any commercial or financial relationships that could be construed as a potential conflict of interest.

## Publisher’s note

All claims expressed in this article are solely those of the authors and do not necessarily represent those of their affiliated organizations, or those of the publisher, the editors and the reviewers. Any product that may be evaluated in this article, or claim that may be made by its manufacturer, is not guaranteed or endorsed by the publisher.
