# Supplementary material for: Prediction of the immunological and prognostic value of five signatures related to fatty acid metabolism in patients with cervical cancer
Source: Front Oncol. 2022 Nov 3;12:1003222. doi: 10.3389/fonc.2022.1003222 (PMC9671136; doi:10.3389/fonc.2022.1003222)
Supplement: Supplementary file 1 [file Table_1.docx]

**Supplementary Table 1 Univariate COX analysis of FAMRGs associated with prognosis in cervical cancer**

| Gene | p.value | HR | Low 95%CI | High 95%CI |
| --- | --- | --- | --- | --- |
| S100A11 | 0.019 | 0.643 | 0.444 | 0.930 |
| SFN | 0.009 | 0.821 | 0.709 | 0.952 |
| GNA14 | 0.016 | 1.543 | 1.085 | 2.195 |
| ENPP1 | 0.002 | 1.759 | 1.231 | 2.514 |
| TBC1D1 | 0.025 | 1.843 | 1.078 | 3.151 |
| TACR1 | 0.004 | 2.648 | 1.365 | 5.136 |
| MYL3 | 0.011 | 4.353 | 1.402 | 13.519 |

Note: FAMRGs: fatty acid metabolism-related genes.
