# Supplementary material for: Prediction of the immunological and prognostic value of five signatures related to fatty acid metabolism in patients with cervical cancer
Source: Front Oncol. 2022 Nov 3;12:1003222. doi: 10.3389/fonc.2022.1003222 (PMC9671136; doi:10.3389/fonc.2022.1003222)
Supplement: Supplementary file 2 [file Table_2.docx]

**Supplementary Table 2 Univariate COX analysis screening for prognostically relevant DEGs in two subtypes**

| Gene | p.value | HR | Low 95%CI | High 95%CI | Gene | p.value | HR | Low 95%CI | High 95%CI |
| --- | --- | --- | --- | --- | --- | --- | --- | --- | --- |
| KRT5 | 0.03988468 | 0.90610759 | 0.82478099 | 0.9954533 | CAPN5 | 0.03924744 | 1.26588489 | 1.0116894 | 1.58394915 |
| CMTM8 | 0.02492581 | 1.49546443 | 1.05201942 | 2.12582945 | TSPAN8 | 0.0023438 | 1.21540922 | 1.07189007 | 1.37814466 |
| TSPAN12 | 0.01331052 | 1.38541626 | 1.07023245 | 1.79342181 | ENTPD8 | 0.04846546 | 1.24462117 | 1.00147607 | 1.54679868 |
| TP63 | 0.0477739 | 0.86190091 | 0.74395659 | 0.99854372 | ABCC3 | 0.0072154 | 1.38919456 | 1.09299438 | 1.76566463 |
| MICALL1 | 0.03726761 | 0.71089682 | 0.51564927 | 0.98007369 | RHPN2 | 0.04587918 | 1.42002266 | 1.00643851 | 2.00356439 |
| RAB17 | 0.02098909 | 1.42498772 | 1.0548829 | 1.92494353 | MUC13 | 0.01434005 | 1.16710578 | 1.03131896 | 1.32077074 |
| DNAJC22 | 0.04800094 | 1.30722917 | 1.00235975 | 1.70482512 | FOXN1 | 0.02952592 | 0.74978896 | 0.57851498 | 0.97176997 |
| RGL3 | 0.04173926 | 1.25894921 | 1.00864911 | 1.57136221 | TMEM98 | 0.02874802 | 1.398976 | 1.03545755 | 1.89011501 |
| TOX3 | 0.00073393 | 1.48738859 | 1.181243 | 1.87287867 | AGR2 | 0.02125965 | 1.16208163 | 1.02264604 | 1.32052896 |
| FAT2 | 0.01048363 | 0.76309811 | 0.62038817 | 0.93863609 | ANG | 0.04364039 | 1.28858874 | 1.0072605 | 1.64849206 |
| KIAA1324 | 0.01654251 | 1.22983407 | 1.03841949 | 1.45653261 | RAB7B | 0.03109906 | 0.72642156 | 0.54323463 | 0.97138191 |
| PLCB4 | 0.00065203 | 1.56679004 | 1.21029555 | 2.02829054 | EPS8L3 | 0.04459172 | 1.20505914 | 1.00451638 | 1.44563847 |
| GM2A | 0.02289228 | 0.67366279 | 0.47935441 | 0.94673491 | FOXA3 | 0.01343967 | 1.2815496 | 1.05271858 | 1.56012196 |
| KLHDC7A | 0.03754645 | 1.31699986 | 1.01598874 | 1.70719276 | PLEKHA6 | 0.02948594 | 1.38787747 | 1.03320728 | 1.8642957 |
| TMC5 | 0.01321724 | 1.26556219 | 1.05045695 | 1.52471516 | RAP1GAP | 0.01379242 | 1.38443909 | 1.06865182 | 1.7935417 |
| SLC2A10 | 0.01375557 | 1.45266484 | 1.0793214 | 1.9551499 | ISYNA1 | 0.03269267 | 1.4105849 | 1.02873172 | 1.93417751 |
| SLC44A4 | 0.04400023 | 1.16776484 | 1.00417648 | 1.35800307 | CGN | 0.0158523 | 1.48094836 | 1.0764113 | 2.03751861 |
| CCDC114 | 0.01069276 | 1.38736205 | 1.07897503 | 1.78389064 | USH1C | 0.02190346 | 1.2658188 | 1.0347412 | 1.54850047 |
| GBP6 | 0.03652722 | 0.82829057 | 0.69421341 | 0.98826277 | LIF | 0.01244626 | 1.47730892 | 1.08784609 | 2.00620443 |
| SH3BGRL2 | 0.04683253 | 1.30399592 | 1.00372543 | 1.69409413 | CST6 | 0.01679463 | 1.27103913 | 1.04419922 | 1.54715734 |
| SELENBP1 | 0.02983974 | 1.28688332 | 1.02495097 | 1.61575404 | KRT15 | 0.01525161 | 0.85064679 | 0.74645202 | 0.96938576 |
| METTL7B | 0.04793574 | 1.27237388 | 1.00219133 | 1.61539542 | LGALS4 | 0.00876588 | 1.20930473 | 1.04910117 | 1.39397225 |
| MLPH | 0.03896853 | 1.24787302 | 1.01127771 | 1.53982141 | SEZ6L2 | 0.0193288 | 1.44480557 | 1.06146219 | 1.96659207 |
| RAB20 | 0.01525681 | 1.50438094 | 1.08164272 | 2.09233787 | SFTA2 | 0.00830095 | 1.18437003 | 1.04452981 | 1.34293187 |
| TSPAN13 | 0.00584987 | 1.59950678 | 1.14530994 | 2.23382497 | SPINK1 | 0.00486321 | 1.29872943 | 1.08269808 | 1.55786565 |
| GOLM1 | 0.02728029 | 1.40040116 | 1.03848189 | 1.8884522 | B3GNT3 | 0.02540153 | 1.28034016 | 1.03090219 | 1.59013235 |
| CALML3 | 0.01264804 | 0.8582218 | 0.76103993 | 0.96781343 | CAMK2N1 | 0.03312507 | 1.27255621 | 1.01948719 | 1.58844497 |
| TMEM125 | 0.02973572 | 1.3223759 | 1.02784403 | 1.70130679 | SERPINB7 | 0.01602592 | 1.28499036 | 1.04778945 | 1.57588934 |
| FBLN5 | 0.0009179 | 2.13249612 | 1.36273001 | 3.33708047 | FAM3D | 0.021176 | 1.21542274 | 1.02962149 | 1.43475292 |

Note: DEGs: Differentially expressed genes.
