# Supplementary material for: Prediction of the immunological and prognostic value of five signatures related to fatty acid metabolism in patients with cervical cancer
Source: Front Oncol. 2022 Nov 3;12:1003222. doi: 10.3389/fonc.2022.1003222 (PMC9671136; doi:10.3389/fonc.2022.1003222)
Supplement: Supplementary file 3 [file Table_3.docx]

**Supplementary Table. 3 Description of key genes**

| Symbol | Description |
| --- | --- |
| PLCB4 | Phospholipase C Beta 4 |
| FBLN5 | Fibulin 5 |
| TSPAN8 | Tetraspanin 8 |
| CST6 | Cystatin E/M |
| SERPINB7 | Serpin Family B Member 7 |
