# Supplementary material for: Prediction of the immunological and prognostic value of five signatures related to fatty acid metabolism in patients with cervical cancer
Source: Front Oncol. 2022 Nov 3;12:1003222. doi: 10.3389/fonc.2022.1003222 (PMC9671136; doi:10.3389/fonc.2022.1003222)
Supplement: Supplementary file 4 [file Table_4.docx]

**Supplementary Table 4 90 pathways with significant differences**

| 90 pathways with significant differences | | |
| --- | --- | --- |
| ABC_TRANSPORTERS ** | DILATED_CARDIOMYOPATHY *** | GLYCOSAMINOGLYCAN_BIOSYNTHESIS_KERATAN_SULFATE *** |
| ACUTE_MYELOID_LEUKEMIA *** | DNA_REPLICATION *** | GLYCOSAMINOGLYCAN_DEGRADATION *** |
| ADIPOCYTOKINE_SIGNALING_PATHWAY * | DORSO_VENTRAL_AXIS_FORMATION * | GLYCOSPHINGOLIPID_BIOSYNTHESIS_GANGLIO_SERIES *** |
| AMYOTROPHIC_LATERAL_SCLEROSIS_ALS *** | DRUG_METABOLISM_OTHER_ENZYMES * | GLYCOSPHINGOLIPID_BIOSYNTHESIS_LACTO_AND_NEOLACTO_SERIES ** |
| APOPTOSIS ** | ECM_RECEPTOR_INTERACTION ** | GNRH_SIGNALING_PATHWAY ** |
| ARRHYTHMOGENIC_RIGHT_VENTRICULAR_CARDIOMYOPATHY_ARVC ** | ENDOCYTOSIS ** | HISTIDINE_METABOLISM *** |
| B_CELL_RECEPTOR_SIGNALING_PATHWAY ** | ENDOMETRIAL_CANCER ** | HOMOLOGOUS_RECOMBINATION *** |
| BASAL_TRANSCRIPTION_FACTORS *** | EPITHELIAL_CELL_SIGNALING_IN_HELICOBACTER_PYLORI_INFECTION *** | HYPERTROPHIC_CARDIOMYOPATHY_HCM *** |
| BLADDER_CANCER *** | ERBB_SIGNALING_PATHWAY *** | INSULIN_SIGNALING_PATHWAY *** |
| CELL_CYCLE *** | FC_EPSILON_RI_SIGNALING_PATHWAY *** | LYSINE_DEGRADATION * |
| CHRONIC_MYELOID_LEUKEMIA *** | FC_GAMMA_R_MEDIATED_PHAGOCYTOSIS *** | MAPK_SIGNALING_PATHWAY * |
| CITRATE_CYCLE_TCA_CYCLE ** | FRUCTOSE_AND_MANNOSE_METABOLISM * | MATURITY_ONSET_DIABETES_OF_THE_YOUNG *** |
| COLORECTAL_CANCER *** | GLIOMA *** | MISMATCH_REPAIR ** |
| COMPLEMENT_AND_COAGULATION_CASCADES *** | GLYCOLYSIS_GLUCONEOGENESIS *** | MTOR_SIGNALING_PATHWAY * |
| CYTOSOLIC_DNA_SENSING_PATHWAY *** | GLYCOSAMINOGLYCAN_BIOSYNTHESIS_CHONDROITIN_SULFATE * | N_GLYCAN_BIOSYNTHESIS *** |
| NATURAL_KILLER_CELL_MEDIATED_CYTOTOXICITY * | PHENYLALANINE_METABOLISM * | SELENOAMINO_ACID_METABOLISM ** |
| NEUROACTIVE_LIGAND_RECEPTOR_INTERACTION ** | PORPHYRIN_AND_CHLOROPHYLL_METABOLISM * | SPHINGOLIPID_METABOLISM ** |
| NEUROTROPHIN_SIGNALING_PATHWAY *** | PRION_DISEASES *** | SPLICEOSOME *** |
| NICOTINATE_AND_NICOTINAMIDE_METABOLISM *** | PROGESTERONE_MEDIATED_OOCYTE_MATURATION *** | STEROID_BIOSYNTHESIS *** |
| NON_SMALL_CELL_LUNG_CANCER *** | PROTEASOME *** | SULFUR_METABOLISM *** |
| NOTCH_SIGNALING_PATHWAY ** | PROTEIN_EXPORT *** | T_CELL_RECEPTOR_SIGNALING_PATHWAY *** |
| NUCLEOTIDE_EXCISION_REPAIR *** | PROXIMAL_TUBULE_BICARBONATE_RECLAMATION ** | TASTE_TRANSDUCTION *** |
| O_GLYCAN_BIOSYNTHESIS *** | PYRIMIDINE_METABOLISM *** | TAURINE_AND_HYPOTAURINE_METABOLISM *** |
| OLFACTORY_TRANSDUCTION ** | REGULATION_OF_AUTOPHAGY * | TERPENOID_BACKBONE_BIOSYNTHESIS * |
| OOCYTE_MEIOSIS *** | RENAL_CELL_CARCINOMA *** | TOLL_LIKE_RECEPTOR_SIGNALING_PATHWAY *** |
| OTHER_GLYCAN_DEGRADATION *** | RENIN_ANGIOTENSIN_SYSTEM *** | UBIQUITIN_MEDIATED_PROTEOLYSIS *** |
| P53_SIGNALING_PATHWAY *** | RIBOFLAVIN_METABOLISM *** | VASCULAR_SMOOTH_MUSCLE_CONTRACTION ** |
| PANCREATIC_CANCER *** | RIG_I_LIKE_RECEPTOR_SIGNALING_PATHWAY ** | VEGF_SIGNALING_PATHWAY ** |
| PATHOGENIC_ESCHERICHIA_COLI_INFECTION * | RNA_DEGRADATION *** | VIBRIO_CHOLERAE_INFECTION * |
| PENTOSE_PHOSPHATE_PATHWAY * | RNA_POLYMERASE *** | WNT_SIGNALING_PATHWAY * |
