# Supplementary material for: Prediction of the immunological and prognostic value of five signatures related to fatty acid metabolism in patients with cervical cancer
Source: Front Oncol. 2022 Nov 3;12:1003222. doi: 10.3389/fonc.2022.1003222 (PMC9671136; doi:10.3389/fonc.2022.1003222)
Supplement: Supplementary file 5 [file Table_5.docx]

**Supplementary Table 5 53 pathways with highly significant differences in different risk groups**

| **pathway** | **p.value** | **sig** |
| --- | --- | --- |
| O_GLYCAN_BIOSYNTHESIS | 2.07E-13 | *** |
| CELL_CYCLE | 1.28E-12 | *** |
| BASAL_TRANSCRIPTION_FACTORS | 2.52E-12 | *** |
| P53_SIGNALING_PATHWAY | 5.53E-12 | *** |
| OOCYTE_MEIOSIS | 8.99E-11 | *** |
| RNA_POLYMERASE | 3.39E-09 | *** |
| UBIQUITIN_MEDIATED_PROTEOLYSIS | 1.46E-08 | *** |
| PROGESTERONE_MEDIATED_OOCYTE_MATURATION | 2.20E-08 | *** |
| RIBOFLAVIN_METABOLISM | 2.58E-08 | *** |
| GLYCOSAMINOGLYCAN_BIOSYNTHESIS_KERATAN_SULFATE | 3.73E-08 | *** |
| BLADDER_CANCER | 6.83E-08 | *** |
| GLYCOSAMINOGLYCAN_DEGRADATION | 8.99E-08 | *** |
| OTHER_GLYCAN_DEGRADATION | 1.28E-07 | *** |
| EPITHELIAL_CELL_SIGNALING_IN_HELICOBACTER_PYLORI_INFECTION | 2.13E-07 | *** |
| PANCREATIC_CANCER | 3.25E-07 | *** |
| NEUROTROPHIN_SIGNALING_PATHWAY | 4.37E-07 | *** |
| HYPERTROPHIC_CARDIOMYOPATHY_HCM | 5.29E-07 | *** |
| NON_SMALL_CELL_LUNG_CANCER | 8.03E-07 | *** |
| N_GLYCAN_BIOSYNTHESIS | 1.50E-06 | *** |
| ERBB_SIGNALING_PATHWAY | 2.18E-06 | *** |
| ACUTE_MYELOID_LEUKEMIA | 2.21E-06 | *** |
| GLIOMA | 3.39E-06 | *** |
| STEROID_BIOSYNTHESIS | 7.52E-06 | *** |
| RENAL_CELL_CARCINOMA | 9.18E-06 | *** |
| CYTOSOLIC_DNA_SENSING_PATHWAY | 9.84E-06 | *** |
| GLYCOLYSIS_GLUCONEOGENESIS | 2.30E-05 | *** |
| FC_GAMMA_R_MEDIATED_PHAGOCYTOSIS | 2.40E-05 | *** |
| GLYCOSPHINGOLIPID_BIOSYNTHESIS_GANGLIO_SERIES | 2.86E-05 | *** |
| SPLICEOSOME | 3.14E-05 | *** |
| TASTE_TRANSDUCTION | 3.90E-05 | *** |
| CHRONIC_MYELOID_LEUKEMIA | 6.58E-05 | *** |
| DILATED_CARDIOMYOPATHY | 8.38E-05 | *** |
| TAURINE_AND_HYPOTAURINE_METABOLISM | 1.16E-04 | *** |
| RENIN_ANGIOTENSIN_SYSTEM | 1.19E-04 | *** |
| MATURITY_ONSET_DIABETES_OF_THE_YOUNG | 1.44E-04 | *** |
| RNA_DEGRADATION | 1.48E-04 | *** |
| SULFUR_METABOLISM | 1.71E-04 | *** |
| PYRIMIDINE_METABOLISM | 1.83E-04 | *** |
| FC_EPSILON_RI_SIGNALING_PATHWAY | 2.07E-04 | *** |
| T_CELL_RECEPTOR_SIGNALING_PATHWAY | 2.41E-04 | *** |
| TOLL_LIKE_RECEPTOR_SIGNALING_PATHWAY | 2.94E-04 | *** |
| INSULIN_SIGNALING_PATHWAY | 3.55E-04 | *** |
| PROTEIN_EXPORT | 3.71E-04 | *** |
| HOMOLOGOUS_RECOMBINATION | 4.83E-04 | *** |
| COMPLEMENT_AND_COAGULATION_CASCADES | 5.36E-04 | *** |
| HISTIDINE_METABOLISM | 5.66E-04 | *** |
| NUCLEOTIDE_EXCISION_REPAIR | 6.32E-04 | *** |
| PRION_DISEASES | 6.59E-04 | *** |
| NICOTINATE_AND_NICOTINAMIDE_METABOLISM | 6.75E-04 | *** |
| COLORECTAL_CANCER | 8.63E-04 | *** |
| AMYOTROPHIC_LATERAL_SCLEROSIS_ALS | 8.89E-04 | *** |
| PROTEASOME | 9.16E-04 | *** |
| DNA_REPLICATION | 9.22E-04 | *** |
